# Social Rhythm and Mental Health: A Cross-Cultural Comparison

**DOI:** 10.1371/journal.pone.0150312

**Published:** 2016-03-08

**Authors:** Jürgen Margraf, Kristen Lavallee, XiaoChi Zhang, Silvia Schneider

**Affiliations:** 1 Clinical Child and Adolescent Psychology, Ruhr-Universität Bochum, Bochum, Germany; 2 Division of Developmental and Personality Psychology, Institute of Psychology, University of Basel, Basel, Switzerland; University of Vienna, School of Psychology, AUSTRIA

## Abstract

**Background:**

Social rhythm refers to the regularity with which one engages in social activities throughout the week, and has established links with bipolar disorder, as well as some links with depression and anxiety. The aim of the present study is to examine social rhythm and its relationship to various aspects of health, including physical health, negative mental health, and positive mental health.

**Method:**

Questionnaire data were obtained from a large-scale multi-national sample of 8095 representative participants from the U.S., Russia, and Germany.

**Results:**

Results indicated that social rhythm irregularity is related to increased reporting of health problems, depression, anxiety, and stress. In contrast, greater regularity is related to better overall health state, life satisfaction, and positive mental health. The effects are generally small in size, but hold even when controlling for gender, marital status, education, income, country, and social support. Further, social rhythm means differ across Russia, the U.S., and Germany. Relationships with mental health are present in all three countries, but differ in magnitude.

**Conclusions:**

Social rhythm irregularity is related to mental health in Russia, the U.S., and Germany.

## Introduction

Just as daily biological patterns, such as circadian rhythm, temperature fluctuations, and cortisol levels, are integral to good mental health, with disruptions associated with depression [1], so it appears are rhythmic social and behavioral patterns, for example in mealtimes, bedtimes, and patterns of social interaction [2,3]. According to the “social zeitgeber” theory (*Zeitgeber* is German for “time–giver”), disruptions in time-cues that trigger the body’s patterns of biological and social behavior may result in increased symptoms and episodes of mental disorder [3,4]. Indeed, some research points to an association between disrupted and irregular social patterns and depressive and bipolar disorder symptoms and episodes [4,5], while other research suggests that rhythm is linked only to bipolar and not unipolar depression [6]. Further, several areas of mental health remain unexplored as they relate to social rhythm, including and especially positive mental health and life satisfaction. Rhythmicity does appear to be important for mental health, especially bipolar depression, yet is understudied (including cross-culturally) and, importantly, is without a brief standard measure by which to quickly and routinely assess it in large-scale studies and screenings.

### Social rhythm and mental health

Low or irregular social rhythmicity appears to be correlationally and causally related to some aspects of negative mental health, in particular to bipolar disorder. Some cross-sectional research indicates that lower trait social rhythmicity is associated with concurrent bipolar disorder in comparison to healthy controls [7], and university students at risk for bipolar depression show less regularity of daily activities and sleep than controls [8]. A review summarizing the small number of existing studies at the time, concluded that disruptive events are associated with bipolar disorder as well as with social rhythm disruption, but that as of the time of the review, the link between social rhythm disruption and mental health was still largely unexplored [4]. Newer prospective longitudinal research indicates that social rhythm irregularity is associated with quicker onset of depressive and manic episodes in bipolar individuals [9], and life events that disrupt regularity are related to depressive and manic symptoms and episodes in people with bipolar disorder [2]. Indeed, people with bipolar disorder are even more susceptible to social rhythm disruption following life events than healthy non-disordered individuals [10], and it is this social rhythm disruption that is hypothesized to be a proximal cause of disrupted mental health [3,4]. Further, people with manic bipolar disorder may be especially vulnerable to episode onset after events that disrupt regularity, and even more vulnerable than people with other types of bipolar disorder or unipolar depression [11]. One other study indicates that rhythm irregularity is related (inconsistently) with affective symptoms, but not with bipolar disorder status [2], and in another study of 15 bipolar individuals and 72 individuals with either high or low vulnerability to bipolar disorder, social rhythm discriminated among high and low vulnerability, but did not differentiate the clinical bipolar group from the other two groups [12]. These last two studies indicate a degree of disagreement among studies of rhythmicity and bipolar disorder. Finally, a recent study of over 7,000 participants from a representative German sample indicates that irregular social rhythm is related to greater depression, anxiety, and stress [13], with small but positive significant effects.

Additional support in favor of the link between rhythmicity and mental health comes from therapy research, which indicates that therapy to increase rhythmicity is effective in treating bipolar disorder [14,15,16,17,18]. Specifically, Interpersonal and Social Rhythm Therapy (IPSRT) targets the maintenance of rhythmic and regular patterns of social behavior and the events that trigger irregularities, with the goal of maintaining regular circadian rhythm and staving off bipolar episodes. Research indicates that bipolar individuals receiving IPSRT increase the rhythmicity of their activities faster than those assigned to the standard clinical management group, stabilize as quickly as controls, and maintain longer episode-free periods during the maintenance phase, with increased regularity associated with decreased disorder recurrence [14]. In another study, intensive social rhythm therapy was as effective as cognitive-behavioral therapy and family-focused therapy, and more effective than collaborative care, in enhancing the effects of pharmacotherapy in treating bipolar disorder [19].

Aside from the research on bipolar disorder, there is little other research on social rhythm and broad mental health and no known cross-cultural comparison. Existing research on rhythmicity and depression yields more mixed results than those for bipolar disorder. Depressed people tend to display less stable social rhythm than non-depressed people [20]. Depressive episodes and/or sleep loss are associated with low social rhythm stability at the time of spousal bereavement in the elderly [21], and in turn further predict higher depression at baseline as well as follow-up in both bereaved and control subjects [22]. Some cross-sectional research also indicates a link between social rhythm irregularity and depression in the elderly, but also finds that social support is either inversely related (in healthy people) or not related (in depressed people) to rhythm, suggesting that social support and activity may be more important than rhythm in depression [6]. Further, it may also be that people with certain disorders, such as unipolar depression, are more prone that non-disordered people to social irregularity, and that this irregularity (and preceding disruptive events) is not necessarily the cause of disorder [4] as it appears to be with manic bipolar disorder [11]. Finally, some evidence points to a relationship between circadian rhythm (sleep-wake cycles and cortisol) and anxiety in one review study [23], as well as social rhythm disruption and anxiety disorders in another empirical study [24].

Other cross-sectional studies do not find the predicted effects of social rhythm or lifestyle regularity on mental health, specifically depression. For example, in one study of 97 adults (majority falling into the “normal” range on the questionnaire measure of depression), while sleep quality was related to loneliness and depression, rhythmicity was not [25]. In a cross-sectional study of university students, those at risk for unipolar depression did not differ from controls in regularity of daily activities and sleep [8]. Finally, in a study of 143 healthy working adults, higher social rhythm regularity was inversely related to minor psychiatric symptoms, but closer examination indicated that this relationship was largely explained by increased activity levels [26].

Finally, no known solid research has investigated rhythmicity and optimal mental states, such as positive mental health or life satisfaction in healthy populations. Very little research has examined social rhythmicity in general populations at all. Some research indicates that intensive psychosocial therapy that includes social rhythm therapy, has positive effects on life satisfaction in bipolar patients [27], but social rhythm therapy was combined with multiple psychosocial therapy modalities in this study, so exact effects are difficult to determine. Research on personality indicates that people with higher trait levels of conscientiousness tend to healthier both physically and mentally [28], and that conscientiousness is related to some aspects of circadian rhythm, such as morningness [29]. Thus, it can be speculated that other aspects of rhythmicity, such as social rhythm, may also be related in general to positive mental health and life satisfaction in general populations. However, this link has not yet been explored. Research on one healthy adult sample indicates that social rhythmicity is associated with older age, sleep quality, and indeed morningness but not necessarily with certain personality traits, such as extraversion or neuroticism [30]. Other aspects of mental health were unexplored in this study. A large-scale analysis of population diary data indicated that people with the highest rhythmicity were those cohabiting with a partner and children, and older people, while single younger people were generally the least rhythmic. Further, high rhythmicity was associated with lower distress, and low rhythmicity was associated with higher social and emotional dysfunction [31].

Finally, there is no known cross-cultural comparison of the relationship between rhythmicity and mental health. Most research to date has been conducted with U.S. and Western European populations. However, there may be some differences across nationalities, based on cultural differences and on differences in the prevalence of mental disorder across countries. For example, some research findings indicate depression prevalence rates are nearly twice as high in the U.S. as in Germany (12-month prevalence of 8.3 versus 3.0%) [32,33], though other city-specific studies have indicated more depression in Mainz, Germany, for example, than in Seattle, U.S. [34]. Research indicates an even greater depression rate in Eastern Europeans, including Russians (point prevalence for one week depression rates in Russian men of 23.1% and in women of 43.9%) [35]. Given their high rates of depression, combined with a transitional societal context with less political and legal stability than in the West, one might expect Russians to have the lowest rhythmicity of all, though, again, cross-national studies on rhythmicity are lacking.

### Assessment metrics

A reliable and valid diagnostic tool is important. The Social Rhythm Metric (SRM) is the primary existing measure of social rhythm and routine. It consists of 17 activities that can be conducted with daily regularity, such as mealtimes, commuting, bedtimes, and television-watching. Activity times are recorded at the end of the day across activities, with regularity calculated as the number of activities (0–17) that were conducted within 45 minutes of the average time for that activity at least three times within a week. Validity research indicates more rhythmicity in controls than in patients, and more “other person prompted” rhythms in patients [36,37]. It discriminates between depressed and non-depressed [20], and between healthy and bipolar people [2]. It has a consistency across two weeks of daily recordings of r = .44 and is correlated positively with other indicators of stability [36,37]; however, as a diary measure, it is very time consuming for participants to complete. The SRM-5 is a short version of the measure which includes items related to getting out of bed, first contact with another person, starting work, dinner, and going to bed [38]. It is generally realiable and valid when compared with the 17-item version, but is still more time consuming to complete, as a diary measure, than a questionnaire would be. Reliable and valid short versions of the diary measure also exist in other languages, such as Portuguese, but again are longer to complete than a questionnaire [39]. There is a strong need for a valid and reliable brief social rhythm measure that can be widely used in large-scale studies with substantial power to detect effects ranging from small to large, to screen for social rhythmicity or that can be used as a quick assessment of this important variable for inclusion to address substantive questions regarding rhythmicity and health.

### The present study

The purpose of the present study was to examine the relationship between social rhythm and mental health using the new Brief Social Rhythm Scale in a large multi-national general-population sample. The BSRS was developed to quickly assess rhythmicity in eating, sleeping, and socializing in large samples and in multiple languages. Based on prior research, we expected that the BSRS would be negatively related to symptoms of depression, anxiety, and a subclinical, yet important risk-factor: stress. We also expected that it would be positively related to positive mental health and life satisfaction. Examination of cross-cultural differences between the fully industrialized US and Germany and people living under the pressure of a transitional society in Russia were considered somewhat exploratory, but based on extant knowledge of disorder prevalence in those three countries, Germany was expected to have the highest rhythmicity, and Russia the lowest. Regular rhythm should be related to better health in all countries.

## Method

### Procedure

Data for the present study was drawn from the BOOM (Bochum Optimism and Mental Health) study, a large-scale, cross-cultural, longitudinal investigation of risk and protective factors in mental health [40,41]. The dataset for the present study is available in S1 Dataset. The Ethics Committee of the Faculty of Psychology of the Ruhr-Universität Bochum approved the study. All national regulations and laws regarding human subjects research were followed, and required permission obtained. Data were collected between November 2012 and February 2014 through three professional opinion research institutes. Four different assessment methods were used in the BOOM study with German representative samples: face-to-face interviews, telephone interviews, online survey, and a mixed-method-approach that allowed individuals to participate either online or via set-top box (a device that allows a person to answer questionnaires via a television and a remote control), and one method was used with representative samples from the USA and Russia: telephone interviews [42]. Participants in the present study were recruited via telephone. Trained professional interviewers at three professional research institutes conducted the telephone interviews with computer assistance. Participants in the present study gave their informed consent orally after being informed about anonymity and voluntariness of the survey. Informed consent had to be given orally, as no written materials were exchanged in the telephone interviews. The interviews were conducted using a CATI (Computer Assisted Telephone Interview) approach. Oral consent was the necessary precondition to start the interview, and no interview could start without it. Interviewers were obliged to obtain oral consent and documented this at the beginning of the CATI data entry mask before the data from the interview questions. The Ethics Committee of the Faculty of Psychology of the Ruhr-Universität Bochum approved this consent procedure. Participants received no financial compensation. Participation took less than an hour at each time point (average of about 45 minutes). Representativeness for the adult residential populations in the three countries was based on the register-assisted census data from 2011 regarding age, gender and education, was ensured via systematized sampling procedures.

### Participants

Participants included 2037 representative members of the German population, 3020 people recruited as a representative sample from Russia, and 3038 people recruited as a representative sample from the USA. In total, 8095 participants completed the survey. Table 1 provides an overview of the sample characteristics, including gender, marital status, educational level and data assessment method. Table 2 contains information about age.

**Table 1:** Demographic information: Numbers of participants by country.

	Germany		Russia		USA		TOTAL
	N	%	N	%	N	%	N
Gender							
Women	1181	25.82%	1607	35.13	1786	39.05	4574
Men	856	24.31	1413	40.13	1252	35.56	3521
Missing	0	0	0	0	0	0	0
Marital Status							
Married or live with partner	1023	22.28	1849	40.27	1720	37.46	4592
Single or live alone	978	32.43	1122	37.20	1006	33.36	3016
Missing	36						487
Education							
Did not graduate high school	392	31.04	265	20.91	606	47.98	1263
Graduated high school	1154	28.35	1665	40.90	1252	30.75	4071
Graduated higher education*	438	19.08	695	30.28	1162	50.63	2295
Missing	53		395		18		466
Total	2037		3020		3038		8095

*(college, university, masters, doctorate)

**Table 2:** Means and results of MANOVA assessing mean differences among countries on measures.

	Germany		Russia		USA					
	mean(se)	N^1^	mean(se)	N^1^	mean(se)	N^1^	F(df1; df2); p-value	GE vs USA	GE vs RUS	USA vs RUS
Age	51.95(0.39)	2007	55.12(0.32)	3038	43.24(0.31)	3020				
Social Rhythm^2^	28.41(0.24)	1602	25.99(0.22)	2680	31.57(0.23)	2826	F(2;6765) = 187.16;<.001	<.001	<.001	<.001
EuroQol VAS	72.6(0.46)	1978	72.18(0.45)	3023	67.51(0.42)	2996	F(2;6765) = 42.31;<.001	.411	<.001	<.001
EuroQol 5D	6.19(0.03)	2000	6.68(0.03)	2956	6.39(0.03)	2999	F(2;6765) = 42.35;<.001	<.001	<.001	<.001
DASS-Depression	2.42(0.08)	1996	4.27(0.09)	2975	3.67(0.07)	2820	F(2;6765) = 90.57;<.001	<.001	<.001	.009
DASS-Anxiety	2.02(0.07)	1991	4.43(0.09)	2976	3.09(0.07)	2934	F(2;6765) = 185.19;<.001	<.001	<.001	<.001
DASS-Stress	4.83(0.1)	1993	6.18(0.09)	2960	5.31(0.08)	2936	F(2;6765) = 49.37;<.001	<.001	.018	<.001
Positive Mental Health	21.90(0.11)	1998	23.35(0.09)	2989	20.92(0.10)	2904	F(2;6765) = 120.57;<.001	<.001	<.001	<.001
Satisfaction with Life	27.09(0.13)	1983	27.24(0.12)	3004	23.55(0.12)	2948	F(2;6765) = 237.66;<.001	.527	<.001	<.001
Social Support	63.47(0.19)	1953	59.09(0.22)	2913	61.23(0.18)	2847	F(2;6765) = 118.73;<.001	<.001	<.001	<.001

^1^ Numbers vary due to missing data.

^2^high scores denote irregularity in rhythm

### Measures

#### Social rhythm

The Brief Social Rhythm Scale (BSRS; S1 Appendix) consists of ten items, which assess the irregularity with which participants engage in basic daily activities during the workweek and on the weekend. As with the SRM, the BSRS assesses waking and bedtimes and breakfast and dinner mealtimes. It also assesses the regularity of time spent with others at work/school and during free time. Unlike longer, prior measures [36,37], it leaves out naptimes and television-watching, as these are considered less important than social time, and not universal. Participants are asked to rate the general regularity of each activity in their lives in general using a scale ranging from 1 (very regularly) to 6 (very irregularly), with high mean scores indicating high irregularity. This measure can be administered at a single time point, rather than requiring a week of daily data to score. Summary scores are the average across all 10 items. BSRS showed a slight positive skewed distribution.

#### Socioeconomic status

Socioeconomic status within country was assessed using income data in Germany and the US, and financial position ratings in Russia, and z-scoring within country (thus each country has a mean score of 0 and standard deviation of 1). Income was assessed on a monthly basis in Germany using a scale ranging from 1 (up to 500 Euros per month) to 11 (4,000 Euros or more per month) Income was assessed on an annual basis in the U.S. using a scale ranging from 1 ($0–9,999 per year) to 12 ($150,000 or more per year). One question was used as a measure of financial position in Russia: “How would you currently rate currently your financial position?” Response options included “rather good,” “average,” and “rather poor.”

#### Quality of health

Overall current quality of health was assessed using the EuroQol (EQ-5D-3L) [43,44,45]. First, participants rated current health status on a scale (EuroQol VAS) ranging from 0 (worst imaginable health) to 100 (best imaginable health). Then, participants rated health in five dimensions (EuroQol 5D) (mobility, self-care, usual activities, pain/discomfort, and anxiety/depression) using 3 levels ranging from no problems to extreme problems. Scores across the five dimensions were summed in the present study, and used in the analyses. Validity of the five dimensions (EuroQol 5D) is indicated by convergence with the EQ-VAS [46] and with WHO-5 and known clinical groups across several countries [47].

#### Depression, anxiety and stress scales

The 21-item short version of the Depression, Anxiety and Stress Scales (DASS-21) [48], appropriate for cross-cultural research [49], assessed symptoms of depression, anxiety and stress as outcome variables of daily stressors. Psychometric properties for the short version are comparable to the 42-item long version [50,51]. The DASS-21 is composed of three 7-item subscales for depressive, anxiety and stress symptoms over the past week. Items are rated on a 4-point likert scale from 0 (*did not apply to me at all*) to 3 (*applied to me very much or most of the time*). Psychometric properties for the short version are shown to be similar to those for the long version, and the scale has been shown to be appropriate for cross-cultural research, with measurement invariance across cultures [49]. In the present study, overall Cronbach’s alpha was *α* = .92 in Germany, .94 in USA, and .93 in Russia. The reliability of each subscale was *α*_*depression*_ = .85; *α*_*anxiety*_ = .80; *α*_*stress*_ = .88 in Germany, *α*_*depression*_ = .89; *α*_*anxiety*_ = .83; *α*_*stress*_ = .85 in USA, and *α*_*depression*_ = .81; *α*_*anxiety*_ = .82; *α*_*stress*_ = .86 in Russia.

#### Positive mental health

Positive mental health was assessed with a 9-item Positive Mental Health scale that was developed by our research team for ongoing studies (P-Scale) [52,53]. The scale assesses positive aspects of health and life experiences (e.g., *I am often carefree and in good spirits*, *I enjoy my life*, *I manage well to fulfill my needs*, *I am in good physical and emotional condition)*. Items are answered on a 4-point likert scale ranging from 0 (*do not agree)* to 3 *(agree)*. Cronbach’s alpha was *α* = 0.89 in Germany, .92 in USA, .85 in Russia, and .89 across all three countries combined. Research indicates that this scale is appropriate for cross-cultural research, based on analyses indicating measurement invariance across cultures [52]. Retest reliability for one month was *r* = 0.73, using the same retest reliability sample used for the BSRS (i.e., subsample of Germans from BOOM study who took the measure online or on paper, but not used in the present study).

#### Life satisfaction

The 5-item Satisfaction with Life Scale (SWLS)[54] assessed global life satisfaction using five items (e.g., “In most ways, my life is close to my ideal”). Items are rated on a scale ranging from 1 (*strongly disagree*) to 7 (*strongly agree*). Scores were averaged across items, with higher scores indicating higher life satisfaction. Research indicates that this scale is adequate for cross-cultural research, based on analyses indicating weak to partial strong measurement invariance across German, Russian, and Chinese samples [52]. Internal consistency in the current sample was α = .84 in Germany, .84 in USA, .77 in Russia, and .82 across all three countries combined.

### Data Analysis and Preparation

For statistical analysis, we used SPSS (version 21) [55]. Internal consistency was computed with Cronbach’s α coefficient. Cronbach’s α > 0.70 indicate acceptable, > 0.80 good, and > 0.90 excellent internal consistency (Kline, 2000). Test-retest-reliability between the first and second BSRS assessments was evaluated by calculating Pearson product-moment correlation coefficients between the BSRS scores across two administrations in the German population of people over 18 years old (N = 610 online, N = 684 paper and pencil, N = 1294 total), which were 5 weeks apart (this is a sample from BOOM, but a different sample than the one used for the primary analyses in the present study). One-way analyses of variance and effect sizes (Cohen’s *d*) were used to assess differences in BSRS scores across cultural groups. Multiple linear regression models were used to evaluate the relationship between BSRS scores and mental health measures, controlling for age, education, gender, and SES. Multiple linear regressions modeled the relationship between two or more explanatory variables and a response variable, by fitting a linear equation to observed data. Significance levels were set at α = 0.05.

## Results

### BSRS psychometric properties

In the German representative telephone data, item-total correlations ranged from r = .25 to r = .54. Cronbach’s Alpha was α = .75 in Germany, .83 in USA, and .82 in Russia, and was .82 across all three countries combined. Test-retest-reliability in a subsample study of 1294 people from Germany from the BOOM study (but not participants used in present study) who took the measure online or in paper and pencil format at time 1 and time 2 (4 weeks later) was r = .70.

### Descriptive statistics and correlations

Descriptive statistics for each measure are presented in Table 2. Tables 3 and 4 present correlations among measures within-country. Correlations indicated that across all cultures, social rhythm correlates negatively with depression, anxiety, and stress, and correlates positively with positive mental health and life satisfaction.

**Table 3:** Correlations among measures within country, with Germany below diagonal, USA above diagonal.

	EuroQol VAS	EuroQol 5D	DASS-Stress	DASS-Anxiety	DASS-Depress	Positive Mental Health	Satisfaction with Life	Social Support	Age	SES Z	Social Rhythm^1^
EuroQol VAS	1	-.530**	-.303**	-.364**	-.356**	.381**	.312**	.244**	-.092**	.099**	-.234**
EuroQol 5D	-.596**	1	.412**	.520**	.495**	-.423**	-.358**	-.290**	.177**	-.171**	.350**
DASS-Stress	-.256**	.312**	1	.754**	.758**	-.396**	-.375**	-.304**	-0.015	-.084**	.299**
DASS-Anxiety	-.392**	.428**	.600**	1	.803**	-.387**	-.327**	-.311**	.044*	-.113**	.309**
DASS-Depress	-.370**	.420**	.629**	.749**	1	-.467**	-.443**	-.405**	.070**	-.116**	.345**
Positive Mental Health	.417**	-.417**	-.461**	-.396**	-.507**	1	.512**	.444**	.083**	.075**	-.291**
Satisfaction with Life	.359**	-.366**	-.371**	-.356**	-.488**	.560**	1	.404**	.059**	.112**	-.258**
Social Support	.259**	-.288**	-.236**	-.294**	-.365**	.445**	.389**	1	0.001	.143**	-.254**
Age	-.222**	.241**	-.147**	0.036	-0.013	0.017	-0.007	-.160**	1	-.098**	.052**
SES Z	.243**	-.245**	-.081**	-.178**	-.208**	.161**	.274**	.161**	-.169**	1	-.074**
Social Rhythm^1^	-.163**	.152**	.169**	.138**	.199**	-.212**	-.207**	-.217**	-0.007	-.150**	1

^1^High scores denote greater irregularity.

* = p<.05,

** = p<.01,

*** = p<.001

**Table 4:** Correlations among measures within country, with Russia below diagonal, total (across all three countries) above diagonal.

	EuroQol VAS	EuroQol 5D	DASS-Stress	DASS-Anxiety	DASS-Depress	Positive Mental Health	Satisfaction with Life	Social Support	Age	SES Z	Social Rhythm^1^
EuroQol VAS	1	-,548**	-,270**	-,352**	-,326**	,406**	,301**	,218**	-,240**	-0,001	-,170**
EuroQol 5D	-,564**	1	,384**	,488**	,454**	-,414**	-,305**	-,265**	,261**	-0,021	,204**
DASS-Stress	-,271**	,381**	1	,721**	,738**	-,372**	-,318**	-,276**	-,030**	,024*	,173**
DASS-Anxiety	-,368**	,452**	,750**	1	,783**	-,326**	-,271**	-,297**	,063**	-0,009	,171**
DASS-Depress	-,274**	,379**	,791**	,751**	1	-,422**	-,399**	-,380**	,031**	-0,016	,227**
Positive Mental Health	,395**	-,451**	-,366**	-,333**	-,391**	1	,534**	,392**	-,030**	-,052**	-,223**
Satisfaction with Life	,199**	-,222**	-,285**	-,221**	-,325**	,501**	1	,350**	,045**	-,032**	-,226**
Social Support	,190**	-,175**	-,222**	-,183**	-,286**	,406**	,323**	1	-,044**	,039**	-,145**
Age	-,446**	,416**	,053**	,157**	,055**	-,196**	0,017	-,072**	1	-0,011	-0,015
SES Z	-,216**	,259**	,191**	,192**	,216**	-,280**	-,316**	-,143**	,162**	1	-0,016
Social Rhythm^1^	-,055**	,096**	,122**	,128**	,148**	-,088**	-,112**	-,066**	-,068**	,086**	1

^1^High scores denote greater irregularity.

* = p<.05,

** = p<.01,

*** = p<.001

#### Between-country differences in means and correlations

In order to properly assess mean differences, all measures with more than one item were tested for measurement invariance in the present sample. In sum, all measures (BSRS, EuroQol 5D, DASS Depression, DASS Anxiety, DASS Stress, PMH, SWLS, and Social Support) tested positive for configural invariance, and full weak invariance (except DASS Stress, which was partial weak invariant). DASS Depression, DASS Anxiety, PMH, and Social Support were full strong invariant. BSRS, EuroQol 5D, DASS Stress, and SWLS were partial strong invariant. This was considered adequate to test for mean differences across countries. The details of the invariance analyses are beyond the scope of this paper, but are available from the authors.

All measures were assessed for significant mean differences across countries. MANOVA indicated between-country differences in the mean values of most measures (Table 2). Follow-up post hoc tests (Tamhane, as there was no homogeneity of variance) indicated significant differences between nearly all country group pairs across measures. Social rhythmicity scores, indicating high irregularity, were highest in the US and lowest in Russia, with Germany in between, meaning participants from the US were the least regular, and Russia the most.

Further, Fisher z-tests comparing the strength of correlations between rhythmicity and outcome variables, indicates between-country differences (Table 5). Irregularity, as denoted by high scores on the BSRS, was generally more strongly related to outcomes for people from the USA than for people from Germany or Russia (i.e., more poor health, stress, anxiety, and depression; less heath, positive mental health, satisfaction with life, and social support). There were fewer differences between Germany and Russia, but irregularity seemed to be more strongly related to fewer positive outcomes in Germany than in Russia.

**Table 5:** Fisher Z-Score comparisons of differences between correlations between BSRS and health across countries.

	Z Scores		
	GE vs USA	GE vs RUS	USA vs RUS
**BSRS with**			
EuroQol VAS	2.36*	-3.49**	-6.83**
EuroQol 5D	-6.79**	1.83	9.99**
DASS Stress	-4.41**	1.54	6.87**
DASS Anxiety	-5.78**	0.33	7.06**
DASS Depression	-5.06**	1.67	7.73**
Positive Mental Health	2.70**	-4.05**	-7.82**
Satisfaction with Life	1.73	-3.11**	-5.62**
Social Support	1.25	-4.88**	-7.09**

High BSRS scores = high irregularity in social rhythm.

Z-critical: 1.96 for p<.05; 2.58 for p<.01.

*p<.05,

**p<.01.

### Multiple regression models predicting mental health

Results from the multi-level models predicting mental health, and controlling for gender, marital status, and education (This was a categorical variable with three levels: no high school graduation, graduated high school, and graduated higher education. It was transformed into two dummy codes with no high school graduation serving as the reference category in the regression.), country (two dichotomous dummy-coded variables, with Germany serving as the reference category), SES z-scores, and social support, are presented in Table 6. As expected, social irregularity (denoted by high scores on BSRS) was positively related to problems in general health (as measured by EQ 5D), stress, anxiety, and depression, and negatively related to positive mental health, life satisfaction, and general good health state as measured by EQ VAS, with generally small but significant effects. In general, health problems were also associated with female gender, being from the USA in comparison with Germany (though people from the USA also reported greater positive mental health), or being from Russia in comparison to Germany. Higher relative SES was associated with higher stress and lower positive mental health. Greater health was related to being married, and being more highly educated, and having greater social support. Likewise, positive health was positively associated with male gender, lower age, being married, being more highly educated, and being from the USA. Overall, effects were generally small to moderate, with the largest effects for social support.

**Table 6:** Standardized regression coefficients (betas) from multi-level models predicting health variables.

	Health Outcomes						
	Health Problems				Positive Health		
Predictors	EQ 5D	Stress	Anxiety	Depression	EQ-VAS	PMH	LS
R^2^	.189	.116	.159	.202	.157	.235	.215
Gender	-.112***	-.095***	-.057***	-.028*	.090***	.072***	-.01
Age	.259***	-.025*	.078***	.045***	-.245***	-.030**	.027*
Marital Status	-.021	-.005	-.033**	-.029*	.041**	.029*	.041***
High school graduate	-.066***	-.056**	-.095***	-.100***	.060***	.017	.015
Bachelor or higher degree	-.103***	-.075***	-.140***	-.133***	.082***	.022	.066***
Country = USA	.125***	.097***	.263***	.164***	.004	.198***	.045**
Country = Russia	.088***	-.025	.100***	.095***	-.135***	-.036*	-.216***
SES z-score	.009	.039**	.020	.009	-.028*	-.077***	-.055***
Social Support	-.215***	-.246***	-.224***	-.325***	.201***	.403***	.340***
Social Rhythm	.180***	.158***	.166***	.192***	-.120***	-.121***	-.120***

Gender, 1 = male, 0 = female. Marital status, 1 = married or living with partner, 0 = single or live alone. For education variables, 1 = graduated high school or 1 = Bachelor or higher degree. For country variables, dummy coded with USA or Russia as reference point. SES z-score = Z-score of income in US and Germany and financial position (3 level) in Russia. Stress, Anxiety and Depression are from the DASS. PMH = Positive Mental Health. LS = Life Satisfaction.

* = p<.05,

** = p<.01,

*** = p<.001.

Social Rhythm: high scores = more irregularity.

## Discussion

The aim of the current study was to examine the relationship between social rhythm and mental health cross-culturally, as well as to present the utility of a new brief social rhythm scale. The BSRS is a reliable brief measure that can be used in large-scale studies to quickly screen for social rhythm or to detect even small effects. Social rhythm patterns, in the present study, are related to mental health in expected directions. That is, greater irregularity (as denoted by high scores on the BSRS) is related to greater reporting of health problems, depression, anxiety, and stress. In contrast, greater regularity is related to greater overall health state, life satisfaction, and positive mental health. The effects are generally small in size, but hold even when controlling for gender, marital status, education, income, country, and social support. According to the raw correlations, the largest effects of social rhythm on mental health were found in the USA. There were also between-country differences in mental health reporting. That is, people from the US, in comparison with Germans, tended to report greater symptoms and strengths overall, as they were higher in both negative and positive mental health. Russians, in comparison with Germans, generally reported more health problems and lower positive mental health. Further, women reported greater health problems than men, in line with other research indicating that women generally report more internalizing and men more externalizing problems [56]. Social support had the largest effects on mental health. This seems intuitive, as social rhythm would seem to depend on having other people to call on (i.e., social support). However, as these results indicate, regularity in accessing the social network is still important, even when accounting for the presence of social support.

The present study had several strengths of note. These include the large sample size with high power to detect even small effects, the cross-national sample, and the battery of health measures that included physical health and positive aspects of mental health, as well as negative aspects of mental health. At the same time, the study had some limitations. First, although the BSRS is reliable and distinguishes among categories of mental health, convergent validity data with another well-established social rhythm measure, such as the Social Rhythm Metric, are missing. It would be ideal to compare self-reported ratings of regularity with more objective measures, such as diary methods, and specifically the SRM-5 [38]. Until validity against the established SRM-5 is established, the present study may be considered preliminary, and the BSRS considered simply a screening instrument. In depth study of social rhythm as a primary variable should utilize well-established measures. Second, the battery of mental health measures could have been more comprehensive, including bipolar symptoms and symptoms of other mental disorder or health. Third, our measure of socioeconomic status was not ideal. It would have been more ideal to use the same measure across countries. Finally, our project aims and hypotheses in this study were somewhat broad, resulting in limited practical clinical utility.

In conclusion, social rhythm irregularity is an important factor in predicting increased disorder and health problems, as well as lower positive aspects of mental and physical health. Further, the BSRS detects these relationships, and is a reliable measure for use in large-scale or screening studies, where participant time is limited. Future studies should consider including the BSRS for a comprehensive picture of mental health and disorder, and should examine its relationship with other aspects of mental health, such as bipolar disorder, and its utility in predicting, for example, the onset of bipolar episodes.

## Supporting Information

S1 Appendix Brief Social Rhythm Scale. (DOCX)

S1 Dataset Dataset used for analyses in present study. (SAV)
