# Supplementary material for: Social Rhythm and Mental Health: A Cross-Cultural Comparison
Source: PLoS One. 2016 Mar 8;11(3):e0150312. doi: 10.1371/journal.pone.0150312 (PMC4783111; doi:10.1371/journal.pone.0150312)
Supplement: S1 Appendix — (DOCX) [file pone.0150312.s001.docx]

S1 Appendix. Brief Social Rhythm Scale

The following statements are related to your life rhythm. You will find a series of statements about various every day activities. Please indicate how regularly you perform each of these activities.

|  |  | *Very regularly* | *Quite regularly* | *Somewhat regularly* | *Somewhat irregularly* | *Quite irregularly* | *Very irregularly* |
| --- | --- | --- | --- | --- | --- | --- | --- |
|  |  |  |  |  |  |  |  |
| 1. | Going to bed Mondays through Fridays |  |  |  |  |  |  |
| 2. | Going to bed on the weekend |  |  |  |  |  |  |
| 3. | Getting out of bed Mondays through Fridays |  |  |  |  |  |  |
| 4. | Getting out of bed on the weekend |  |  |  |  |  |  |
| 5. | Meeting other people at school or work Mondays through Fridays |  |  |  |  |  |  |
| 6. | Meeting other people at school or work on the weekend |  |  |  |  |  |  |
| 7. | Meeting other people in my free time Mondays through Fridays |  |  |  |  |  |  |
| 8. | Meeting other people in my free time on the weekend |  |  |  |  |  |  |
| 9. | Taking meals regularly Mondays through Fridays |  |  |  |  |  |  |
| 10. | Taking meals on the weekends |  |  |  |  |  |  |
